# Subjective discomfort and lack of volitional drive with neuroleptic pharmacotherapy - a phenomenological case study

**DOI:** 10.1192/j.eurpsy.2024.1729

**Published:** 2024-08-27

**Authors:** L. Korošec Hudnik, I. Kosmačin

**Affiliations:** ^1^Department for Intensive Psychiatric Treatment, University psychiatric clinic Ljubljna, Ljubljana, Slovenia

## Abstract

**Introduction:**

In comparison to extrapyramidal and metabolic side effects, the subjective aspects of neuroleptic treatment have been less extensively researched. Nevertheless, they are equally significant given their potential to influence adherence and functional outcome. Historically, terms such as “neuroleptic dysphoria,” “neuroleptic-induced psychic indifference,” and “neuroleptic-induced deficit syndrome” were used to characterize a range of unpleasant mood states on the one hand and a documented and observable motivational deficit on the other. The latter aligns with the findings from preclinical neuroscientific studies and animal models highlighting the significant involvement of mesolimbic dopamine in motivational processes. Despite an abundance of anecdotal data these adverse effects are often undetectable in large-scale clinical studies that utilize standardized assessment measures.

**Objectives:**

To present adverse subjective changes in arousal, mood and volitional drive resulting from neuroleptic intake from a patient’s perspective.

**Methods:**

The subject is a patient, with no reported negative symptoms or lasting functional impairment described, who underwent a gradual 6-month discontinuation of risperidone in an outpatient setting following a complete recovery after a single psychotic episode. A semi-structured interview modelled after The Clinical Assessment Interview for Negative Symptoms (CAINS) was conducted. We aimed to elicit descriptions of the subjective experience while ensuring our approach remained non-suggestive.

**Results:**

In addition to describing potential akathisia and lethargy at higher doses, the subject reported a significant lack of motivation and a notably reduced willingness to exert effort towards achieving specific goals or engaging in activities that he still found rewarding or pleasurable. Furthermore, he consistently noted gradual improvements across various psycho-social aspects following the discontinuation of the medication. These adverse and unpleasant experiences were presented as the primary reason for wanting to discontinue pharmacological treatment.

**Conclusions:**

Certain adverse effects of antipsychotic medications can only be elucidated by the clinician through the examination of the patient’s subjective experiences. Medication induced dysphoria and volitional deficits have the potential to profoundly impact treatment adherence, leading to unrecommended discontinuation of neuroleptics, and can cause important functional impairment.

**Disclosure of Interest:**

None Declared

